# Novel Metallo-β-Lactamase *bla*_CVI-1_ Isolated from a *Chromobaterium violaceum* Clinical Strain Resistant to Colistin

**DOI:** 10.3390/pathogens12070961

**Published:** 2023-07-21

**Authors:** Sonia A. Gomez, María Belén Sanz, Melina Rapoport, Graciela Sucin, Teresa A. Corallo, Tomás Poklepovich, Josefina Campos, Paola Ceriana, Juan Manuel de Mendieta, Mónica Prieto, Fernando Pasteran, Alejandra Corso

**Affiliations:** 1 Servicio Antimicrobianos, INEI-ANLIS “Dr. Carlos G. Malbrán”, National and Regional Reference Laboratory in Antimicrobial Resistance (NRRLAR), Buenos Aires C1282AFF, Argentina; 2 Consejo Nacional de Investigaciones Científicas y Técnicas (CONICET), Buenos Aires C1425FQB, Argentina; 3 Sector de Bacteriología, Hosp. Pediátrico “Dr. Avelino Castelán”, Resistencia H3508, Argentinaterecorallo@yahoo.com.ar (T.A.C.); 4 Plataforma de Genómica y Bioinformática, INEI-ANLIS “Dr. Carlos G. Malbrán”, Buenos Aires CP1281, Argentina; 5 Servicio Bacteriología Especial INEI-ANLIS "Dr. Carlos G. Malbrán", Buenos Aires CP1281, Argentina

**Keywords:** *Chomobacterium violaceum*, carbapenemase, metallo-beta-lactamase, colistin resistance, CVI-1

## Abstract

Objective: We aimed to describe a colistin (COL)-resistant (R) *Chromobacterium violaceum* (Cvi) isolate from a septic patient in Argentina expressing a previously unknown gene, *bla*_CVI-1_. Methods: In 2019, a 12 year old child was injured with a thorn in a lagoon. The child was hospitalized due to sepsis and multiple abscesses. Cvi was isolated from skin and soft tissue and tracheal aspirate. The patient was successfully treated with imipenem (IMI) plus amikacin. Antimicrobial susceptibility was assessed by disk diffusion, broth microdilution, and the E-test. Carbapenemase activity was assayed by double-disk synergy and microbiological tests. Resistance, virulence, and additional gene searches were performed by in silico analysis of sequences obtained by whole-genome sequencing (WGS). A maximum likelihood phylogenetic tree was built with public Cvi genomes. Results: R was seen for IMI and COL. Expression of a metallo-β-lactamase was confirmed. Genome analysis revealed *bla*_CVI-1_, a subclass B2 metallo-β-lactamase with 62.66% ID with CphA from *A. hydrophila* (WP081086394). R to COL could be attributed to the *arnC* and *arnT* genes. Virulence factors required for invasion and toxicity were also found. No plasmids were detected. The phylogeny tree showed two main clades with geographical distinction, and the isolate studied here stands alone in a branch closely related to two clinical isolates from the USA. Conclusions: This is the second report of infection by Cvi in Argentina. This pathogen carried a new gene, *bla*_CVI-1_, a metallo-β-lactamase that can be detected by routine methods. Prompt suspicion of *C. violaceum* infection is crucial to treating this rare pathogen rapidly and properly.

## 1. Introduction

*Chromobacterium violaceum* is a facultative anaerobic Gram-negative beta-proteobacterium belonging to the *Neisseriaceae* family. This rod-shaped, motile, non-fastidious, non-sporing bacterium is fermentative and positive for catalase and oxidase. It is predominantly found in the water and soil of tropical and subtropical regions [1]. While *C. violaceum* is rare as a human pathogen, it is capable of causing life-threatening diseases such as respiratory and gastrointestinal infections, urinary tract infections, liver abscesses, meningitis, endocarditis, hemophagocytic syndrome, and fulminant sepsis [1]. There is no age or gender preference for infection, although chronic granulomatous disease and glucose-6-phosphate dehydrogenase deficiency are known predisposing conditions for *C. violaceum* infection [1]. The major sources of infection are cutaneous trauma or injury, as well as ingestion of contaminated seafood or water [1].

*C. violaceum* was first described over a century ago, but it was first isolated in 1976 in Brazil. Subsequent research has primarily focused on its biomedical and biotechnological properties, particularly its metabolite, violacein [1,2]. In 2003, the genome of *C. violaceum* was sequenced, revealing its adaptability to the environment and the presence of virulence and pathogenicity islands that contribute to its ability to cause severe disease in humans [3]. Furthermore, *C. violaceum* demonstrates resistance to several antibiotics such as rifampin, ampicillin and cephalosporins, which complicates the treatment of infections [4,5]. In this regard, therapeutic or susceptibility testing interpretation guidelines are still not available. Consequently, broad-spectrum antibiotics such as carbapenems or fluoroquinolones are used as empirical choices to control *Chromobacterium* infections based on reported clinical outcomes [1].

Previous studies have reported intrinsic resistance mechanisms towards β-lactam antibiotics in *C. violaceum*, particularly evident in its resistance to penicillins and cephalosporins [5,6]. Through complete genome sequencing, the presence of an *ampC* β-lactamase gene, similar to that found in *Pseudomonas aeruginosa*, has been identified in *C. violaceum* [5]. Additionally, the genome revealed the presence of various β-lactamase precursors, including a probable B2 class metallo-β-lactamase [5]. B2-type metallo-β-lactamases exhibit a phylogenetic relationship with the B1 subclass enzymes, which include enzymes such as NDM-1, IMP-1, and VIM-2 [7]. However, B2 enzymes are distinct in their high degree of substrate selectivity within the metallo-β-lactamase group, specifically demonstrating effective inactivation of carbapenems [7]. In addition, the B2-enzymes constitute the smallest Class B subgroup, of which the best-known member is CphA.

To the best of our knowledge, about 160 infections caused by *C. violaceum* were reported worldwide, with twenty-five cases documented in Latin America, including one case in Argentina [8]. In this study, we fully characterized a colistin-resistant *C. violaceum* isolate obtained from a pediatric patient that expressed a novel metallo-β-lactamase named *bla*_CVI-1_.

## 2. Materials and Methods

### 2.1. Identification, Susceptibility Testing, and Microbiological Tests and PCRs

Bacterial identification was performed by Maldi TOF (Bruker, London, UK). Susceptibility testing was determined by disc diffusion following protocols established by CLSI [9], microdilution (MicroScan Walkaway), and/or the epsilometric test (bioMérieux, Buenos Aires, Argentina). As there is no standardization for the interpretation of the susceptibility testing for this microorganism, the results were interpreted considering the breakpoints for Enterobacterales of CLSI M100 [9]. Carbapenemase activity was assayed with the double disc synergy test (meropenem-EDTA-imipenem), the Triton Hodge Test (THT) with meropenem, and the modified Carbapenem Inactivation Method (mCIM and eCIM) following the protocols and interpretation criteria in CLSI 2019 [9]. Additionally, the Blue Carba Test (BCT) and Carba NP (CNP) were also tested following standardized protocols [9,10].

PCRs were performed using the primers listed in Supplementary Material Table S1 to detect the resistance genes: *bla*_PER_, *bla*_CTX-M_, *bla*_KPC_, *bla*_CMY 2/7_, *bla*_VIM_, *bla*_IMP_, *bla*_NDM_, and *mcr-1*.

### 2.2. Whole Genome Sequencing (WGS) and Analysis

Genomic DNA was extracted using the QIACube DNAMini Kit (Qiagen, Germantown, MD, USA). WGS was performed on *C. violaceum* using the Nextera XT DNA library preparation kit and the Illumina MiSeq Platform (Illumina, San Diego, CA, USA) to generate 250 bp paired-end reads. Quality measurements and trimming were performed by FastQC and Trim Galore. Reads were de novo assembled using Unicycler and confirmed for species with Kraken. Automated annotation was performed with Prokka [11]. Average nucleotide identity (ANI) values were determined using the ANI calculator. Genome analysis was performed using the Comprehensive Genome Analysis tool offered by Patric 3.6.8 [12]. Additionally, other bioinformatics tools were used to search for or compare specific genes, genomic regions, or amino acid sequences, like BLASTn and UniProt [13]. Several tools were used directly from the Center for Genomic Epidemiology (https://www.genomicepidemiology.org/last, accessed on 1 June 2023) to search for the presence of insertion sequences: ISfinder; resistance genes, ResFinder, and ARM Finder Plus; and for plasmids, PlasmidFinder. The search of putative genes that could have conferred colistin resistance was performed in the automatic annotation by RAST2 and additional in silico searches (search words: colistin, polypeptide, phospholipid, biosynthesis, and lipopolysaccharide).

### 2.3. Phylogenetic Analysis of C. violaceum M23796

Phylogenetic analysis of *C. violaceum* M23796 was constructed with a WGS dataset of 1 reference strain (ATCC 12472, GCA_000007705) and 29 *C. violaceum* sequences downloaded from Patric 3.6.8. or the NCBI Genome Browser (https://www.ncbi.nlm.nih.gov/genome/?term=Chromobacterium+violaceum, accessed on 5 June 2023). Metadata details of the strains used to build the tree can be found in the Supplementary Material (Table S2). The assemblies were annotated using Prokka v1.5 to achieve uniformity within the dataset, and then Roary was used to generate a core-gene alignment (concatenated genes present in ≥99% of the genomes with ≥95% nucleotide identity). Subsequently, an alignment of 2951 core genes was used to generate a single-nucleotide polymorphism (SNP) alignment with SNP-sites v2.3.2 and to construct a maximum likelihood phylogenetic tree with RAxML v8.2.8 under the generalized time reversible model (GTRCAT) and bootstrapping with 1000 replicates. FastBAPS was used to cluster sequences in the *C. violaceum* phylogeny, which provides a Bayesian hierarchical clustering of the data [14]. The alignments obtained to build the tree were used as input for FastBAPS [14]. The tree was loaded into Microreact [15], a web application to visualize it with geographical and associated metadata (https://microreact.org/project/qyijLM4sQvt5jDLP9gWfah-raxml-cvim23976, accessed on 1 June 2023).

*bla*_CVI-1_ was submitted to the NCBI submission portal (https://submit.ncbi.nlm.nih.gov/). A phylogenetic tree was built by aligning the amino acid sequences of representative metallo-β-lactamase genes known to date downloaded from RefSeq (Table S3). Alignments were performed with the ClustalW program built into MEGA6 [16]. A phylogenetic reconstruction tree was obtained using the neighbor-joining method, tested with 1000 bootstrap replicates, and an amino acid substitution model (Poisson) and evolutionary analysis were conducted in MEGA6. Aligned sequences were seen with FigTree 1.4.4.

### 2.4. Data Availability

*bla*_CVI-1_ MN918151, *C. violaceum* M23796 genome JAHWXW000000000; BioProject PRJNA747180; and BioSample SAMN20283044.

## 3. Results and Discussion

A 12 year old girl, a native of a rural area in the Chaco Province, Argentina, who was previously healthy, was wounded by a thorn in her left foot while bathing in a lagoon. After 24 h, she developed a fever, cellulitis in the ankle and knee, ipsilateral inguinal adenitis, and abdominal pain. The patient was immediately hospitalized at the peripheral health center and treated with ceftriaxone and clindamycin, but there was no positive response. On the 6th day after the injury, she was referred from the local facility to the city hospital, where she was admitted with a fever, hypoxemia, and hemodynamic compromise.

Abscesses were observed in the left ankle, with a superficial blister containing blackish content, purplish maculopapules measuring 0.5 cm that rapidly evolved into blisters, and inguinal adenitis adhering to deep planes. Additionally, macules of less than 2 mm that evolved into micro-abscesses with central necrotic ulceration were observed. The bone scan of the chest showed bilateral interstitial infiltrates. The laboratory results revealed 19,230 leukocytes (1% metamyelocytes, 1% myelocytes, 6% arch neutrophils, and 78% polymorphonuclear cells); Hb 9.6 mg/dL; CRP 317 IU/mL; albumin 2.8 mg/dL; urea 0.6 g/L; and creatinine 1.21 mg/dL. The condition was assumed to be sepsis due to its dermal, joint, and pulmonary focus. Subsequently, the patient was admitted to the ICU due to septic shock, where she required inotropes and mechanical ventilation. The patient was empirically treated with vancomycin, clindamycin, and ceftriaxone. *C. violaceum* was isolated from tracheal aspirate as well as from skin and soft tissue abscesses 48 h after the initiation of empirical treatment. Subsequently, the treatment was switched to imipenem plus amikacin. Immediately, the isolate was referred to the NRRLAR for further study. Eventually, the patient improved and was extubated. However, the patient subsequently experienced daily fever peaks for 17 days, abdominal pain, and worsening of the left inguinal adenitis with abscessed nodules. A new series of blood cultures and abscess samples from para-vesicular, inguinal, and bone were negative. Surgical toilet procedures were performed in the bone and inguinal abscesses, in addition to draining the abdominal abscess. After completing 24 days of treatment with imipenem plus amikacin, the patient finally improved and continued with oral doses of ciprofloxacin and trimethoprim-sulfamethoxazole. After 37 days of hospitalization, the patient was discharged in good health, with the abscesses continuing to heal while receiving levofloxacin [17].

M23796 was confirmed as *C. violaceum* by Maldi TOF. Susceptibility testing for clinically relevant antimicrobials was performed using disc diffusion and broth microdilution methods (see Table 1). Both methods indicated non-susceptibility to ampicillin, ampicillin/sulbactam, amoxicillin/clavulanic acid, and cefotaxime. Discordant results between the two methodologies were observed for imipenem, ceftazidime, cefepime, cefoxitin, and aztreonam, where susceptibility was shown by disk diffusion but non-susceptibility was observed by broth microdilution. Imipenem was also tested using the epsilometric method and was interpreted as intermediate (2 µg/mL) [9]. Meropenem and ertapenem were susceptible according to both methods, as were other therapeutically available antimicrobial classes such as aminoglycosides, sulfonamides, and glycylcyclines, among others. Colistin was confirmed as resistant by broth microdilution.

Considering the non-susceptibility to imipenem, we initially investigated the presence of an enzymatic mechanism of β-lactam resistance using phenotypic methods. Positive synergy was observed between imipenem and meropenem disks with EDTA. The THT method showed growth at the intersection of the streak and the zone of inhibition (Figure S1a). Furthermore, mCIM (7 mm) confirmed the presence of a carbapenemase (Figure S1b), and eCIM (22 mm) confirmed the presence of a metallo-β-lactamase, as per CLSI [9]. The BCT and Carba NP tests yielded indeterminate results due to the intrinsic color displayed by this species. Additionally, all PCR assays conducted to detect locally relevant acquired *bla* genes returned negative results. Taken together, these findings indicated the presence of an unusual metalloenzyme; therefore, we fully sequenced *C. violaceum* M23796.

The whole genome sequencing yielded high-quality reads. The Unicycler assembly rendered 60 contigs, with a genome length of 4,701,626 bp, a GC content of 65%, and 4643 coding DNA sequences (CDSs). Kraken2 confirmed the Maldi TOF result for *C. violaceum.* Additionally, the ANI calculation showed a value of 98.58% for *C. violaceum* ATCC 12472, further supporting the identification. Plasmids were not detected in this isolate. Full details on the sequencing outcome can be found in Supplementary Material Table S4.

**Figure 1:**
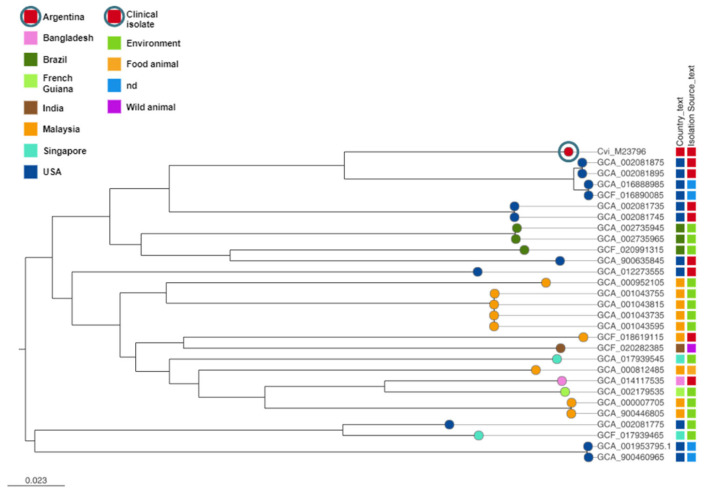
Pan-genome analysis of *C. violaceum*. Tree construction details and analysis are described in the text. Branch tips are color-coded according to the country of isolation, as indicated by the lateral-colored bar. The circled leaf node represents the isolate studied here (Cvi_M23796). Country and isolation sites are labeled with a color code, as shown in the figure. Clusters were determined using FastBAPS based on an alignment of core genes. The scale bar represents the number of mutations per variable site. Full metadata of the isolates can be viewed at (https://microreact.org/project/qyijLM4sQvt5jDLP9gWfah-raxml-cvim23976, accessed on 13 June 2023). “nd” indicates “not determined”.

A phylogenetic tree was constructed using publicly available genomes obtained from animal farms, clinical isolates, freshwater, plants, and soil collected from seven countries spanning two continents (Figure 1; access the Microreact project at (https://microreact.org/project/qyijLM4sQvt5jDLP9gWfah-raxml-cvim23976, accessed on 1 June 2023). The tree topology reveals two main clades: one primarily comprising isolates obtained from the American continent, and another clade consisting of isolates from Asia and the American continent, including India, Malaysia, and French Guiana. The isolate described in this study stands alone in a branch but shares close relatedness to isolates obtained in the USA (Figure 1).

In order to explain the phenotypic and microbiological results, the genome was examined for a potential β-lactamase gene. Consequently, we identified a *cphA*-like gene encoding a subclass B2 β-lactamase. The *cphA*-like gene was submitted to NCBI and designated as *bla*_CVI-1_ (840 bp, 279 aa), which stands for *Chromobacterium violaceum* imipenemase. The amino acid sequence alignment of CVI-1 showed 62.66% identity with CphA from *A. hydrophila* (WP081086394). Furthermore, we investigated conserved residues that are typically found in B2 enzymes. Our analysis confirmed the presence of key conserved residues, including Glu69, Gly84, and Asn220, which are essential for the catalytic activity of these enzymes. Additionally, the presence of one Zn2+ binding site (Asn116, His118, and His196) was identified, consistent with previous reports [7]. Notably, instead of the typical cysteine residue (Cys221), a lysine residue was found at that position. B2 metalloenzymes are characterized by their NHH/DCH active site motif, corresponding to the Zn1 and Zn2 sites, respectively; however, only one zinc ion, in the Zn^2^ site, is required for catalysis [7]. Consequently, CVI-1 still exhibited carbapenem hydrolysis capacity, although further studies are needed to fully understand its enzymatic activity.

**Figure 2:**
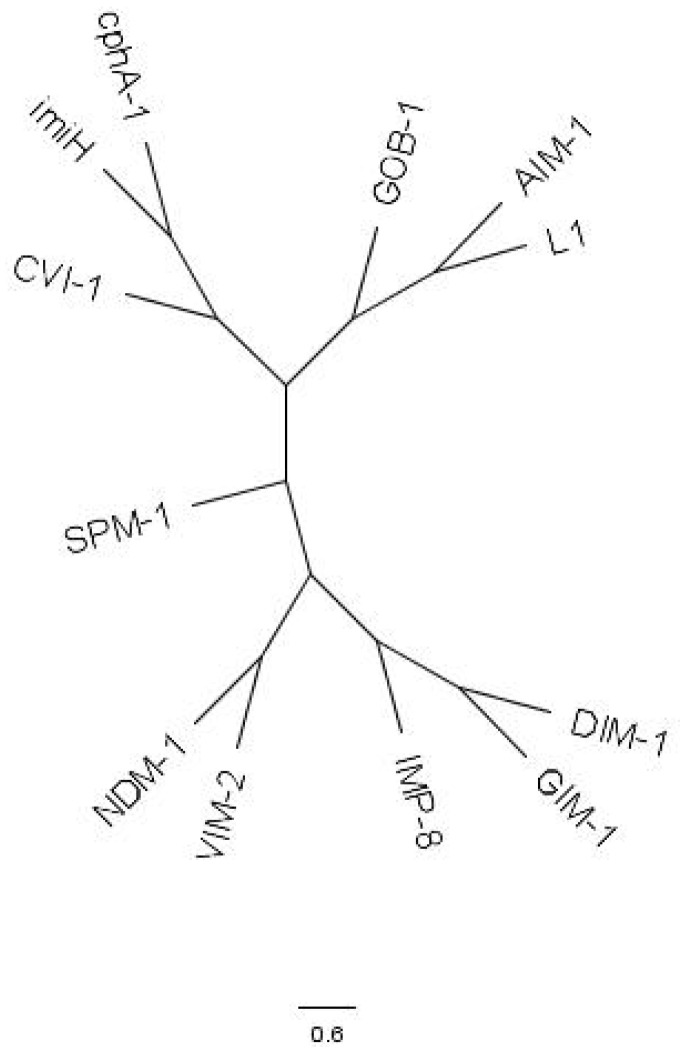
A phylogenetic tree showing the evolutionary relationship between CVI-1 and known Ambler class B β-lactamases is shown. The tree was drawn to scale, and the evolutionary distances are in units of *arnC* amino acid substitutions per site.

To determine the evolutionary relationship of CVI-1 with other Ambler class B metallo-β-lactamases, a phylogenetic tree was constructed (Figure 2). The analysis revealed that CVI-1 forms a distinct monophyletic branch closely related to two other B2 metallo-β-lactamases, CPHA and IMIH. This finding highlights the evolutionary lineage of CVI-1 within the diversity of metallo-β-lactamases. Previous studies found that Subclass B1 + B2 share an evolutionary origin distinct from that of the B3 subclass, although the β -lactam-hydrolysing function evolved independently within each group [7,18]. The identification and characterization of *bla*_CVI-1_ as a subclass B2 metallo-β-lactamase in *C. violaceum* contribute to our understanding of the genetic mechanisms underlying β-lactam resistance in this pathogen. Future studies focusing on the functional and structural characterization of *bla*_CVI-1_ will help elucidate its role in conferring resistance in *C. violaceum* infections.

Additionally, we found a 1191 bp sequence corresponding to an uncharacterized Class C β-lactamase, exhibiting 71% nucleotide identity with *bla*_PDC-67_ from *Pseudomonas aeruginosa* (GenBank NG_049942.1) and 59% amino acid identity with an AMP-C from *Pseudomonas fluorescens* (UniProt P85302).

The search for the genetic basis of colistin resistance revealed two glycosyltransferase family proteins that showed homology, as determined by computational analysis, to *arnC* and *arnT*. Both genes exhibited 100% identity and coverage with only four entries, including ATCC 12472. The gene, previously known as *pmrF* or *yfbF*, is referred to as “Polymyxin resistance protein ArnC, glycosyl transferase (EC 2.4.-.-)”. ArnC functions as an undecaprenyl transferase involved in the biosynthesis pathway of lipopolysaccharides, which is crucial for bacterial outer membrane biogenesis. It acts in a pathway that modifies lipid A phosphates with 4-amino-4-deoxy-L-arabinose (L-Ara4N), leading to increased resistance to polymyxins [19]. Another gene found was *arnT*, previously known as *pmrK* or *yfbI*, and it is designated as “Undecaprenyl phosphate-alpha-4-amino-4-deoxy-L-arabinose arabinosyl transferase (EC 2.4.2.43)”. This protein catalyzes the transfer of the L-Ara4N moiety from the glycolipid undecaprenyl phosphate-alpha-L-Ara4N to lipid A. The modified arabinose is attached to lipid A, and it is essential for resistance against polymyxin and cationic antimicrobial peptides [20]. Therefore, we propose that colistin resistance in *C. violaceum* M23796 may be attributed to the expression of these genes. These findings support the fact that treatment of *C. violaceum* infections is challenging owing to its resistance against different antibiotics, including colistin [21].

In addition, we detected several efflux systems responsible for the unidirectional pumping of cytotoxic drugs into the extracellular environment. The identified genes represent most classes of efflux pumps already described in *C. violaceum* ATCC 12472 [3,5], including macrolide efflux proteins (e.g., MacA and MacB), major facilitator (MF) type efflux pumps (e.g., EmrAB-TolC, MdfA/Cmr) that confer protection against various unrelated antibiotics, and resistance nodulation cell division (RND) pumps (e.g., MdtABC-TolC). Furthermore, we identified resistance gene homologues to aminoglycoside N(6′)-acetyltransferase. The complete list of genes involved in antimicrobial resistance can be found in Table S5.

Due to the rapid clinical course of chromobacteriosis, inappropriate antimicrobial therapy is considered a significant predisposing risk factor in *C. violaceum* infections. In the case presented here, the patient was successfully treated with imipenem plus amikacin during the acute phase of the infection. Subsequently, the patient continued with oral doses of ciprofloxacin plus trimethoprim-sulfamethoxazole and finally with levofloxacin.

Considering the degree of invasion and tissue damage described in the clinical case, we conducted a search for virulence genes that could explain the lesions suffered by the patient. We identified twenty ORFs encoding components of the Type III secretion system (T3SS), all located on the same contig (Table S5). The T3SS functions as a molecular syringe, allowing the secretion of effector molecules that induce cytoskeletal rearrangements in the host cell. It is known to be closely associated with the infection of both animal and plant cells [3].

Furthermore, we detected several virulence genes that are essential for invasion and toxicity, including *spaQ*, *sicA*, *spvC*, *recA*, *hfq*, *pnp*, and *fba* (Table S5). Additionally, seven ORFs were found to encode putative hemolysin genes. Hemolysins are toxins that exhibit hemolytic activity towards mammalian cells and act by forming pore-like structures upon contact with mammalian cells.

## 4. Conclusions

This study presents the second reported case of *C. violaceum* infection in Argentina. The bacterium was isolated from a 12 year old child who suffered a foot injury from a thorn while bathing in a lagoon in the warm province of Chaco, located in the northeast region of Argentina. The patient exhibited advanced signs of infection and tissue damage, requiring hospitalization due to sepsis. Here, we comprehensively characterized a colistin-resistant isolate that carried a previously unidentified β-lactamase gene named *bla*_CVI-1_. The presence of this gene, along with other antimicrobial and virulence determinants, highlights the versatility and dangerous nature of this pathogen, capable of causing severe disease. Continuous surveillance is essential to monitor the spread of this hazardous pathogen and implement appropriate measures for its control.

## Figures and Tables

**Table 1:** Antimicrobial susceptibility of *C. violaceum* M23796.

	Disc Diffusion		MICs	
Antimicrobial Agent	mm	Int.	µg/mL	Int.
Imipenem	23	S	4 *	R
Meropenem	32	S	≤1	S
Ertapenem	29	S	0.5	S
Ampicillin	6	R	32	R
Ampicillin/Sulbactam	11	R	>16	R
Amoxicillin/Clavulanic Acid	6	R	>16	R
Ceftazidime	22	S	>16	R
Ceftazidime/Clavulanic Acid	20	na	>2	na
Ceftazidme/Avibactam	30	S	nd	na
Ceftolozano/Tazobactam	30	S	nd	na
Cefepime	28	S	16	R
Cefotaxime	21	R	>16	R
Cefotaxime/Clavulanic acid	19	na	>4	na
Cefoxitin	21	S	>16	R
Aztreonam	21	S	>8	I
Piperacillin/Tazobactam	29	S	≤16	S
Trimethoprime/Sulfamethoxazole	30	S	≤2	S
Amikacin	20	S	≤16	S
Gentamicin	21	S	≤4	S
Ciprofloxacin	40	S	≤1	S
Fosfomycin	16	na	≤64	na
Minocycline	30	S	nd	na
Tigecycline	29	na	≤1	na
Colistin	6	na	>4	R

* MIC performed by MicroScan Walkaway microdilution and Etest (2 µg/mL). nd, not determined; na, not available; Int., interpretation (M100 CLSI).

## Data Availability

*bla*_CVI-1_ MN918151; *C. violaceum* M23796 genome JAHWXW000000000; BioPorject PRJNA747180; and BioSample SAMN20283044.

## Supplementary Materials

The following supporting information can be downloaded at: https://www.mdpi.com/article/10.3390/pathogens12070961/s1, Figure S1a. THT, Figure S1b, mCIM, and eCIM of *C. violaceum* M23796; Table S1. Primers used; Table S2. Metadata of the phylogenetic tree of *C. violaceum* isolates; Table S3. Unicycler assembly outcome of *C. violaceum* M23796; Table S4. Metadata of MBL phylogeny tree construction; Table S5. *C. violaceum* M23796 AMR and virulence genes.
