# Supplementary material for: Novel Metallo-β-Lactamase blaCVI-1 Isolated from a Chromobaterium violaceum Clinical Strain Resistant to Colistin
Source: Pathogens. 2023 Jul 21;12(7):961. doi: 10.3390/pathogens12070961 (PMC10385785; doi:10.3390/pathogens12070961)
Supplement: Supplementary file 1 [file pathogens-12-00961-s001.zip › pathogens-2476972-supplementary for XML final.pdf]

Phenotypic characterization of *Chromobacterium violaceum* M23796

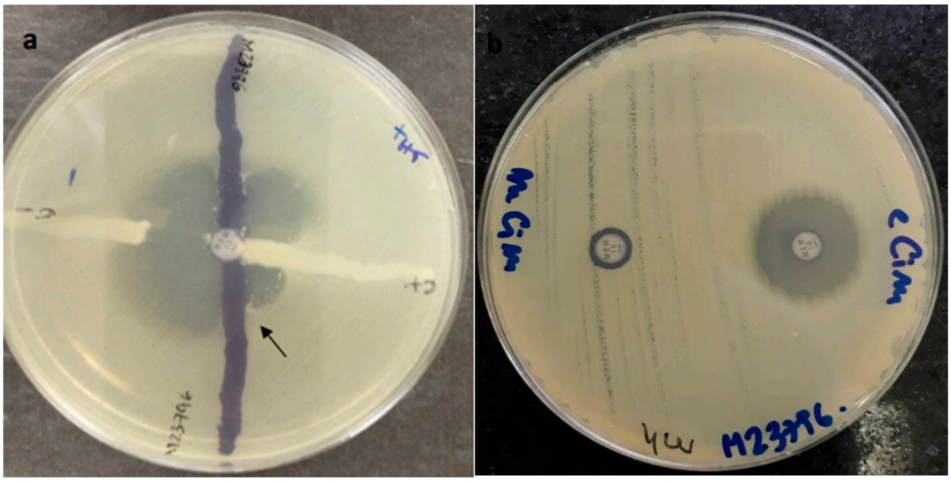

Figure S1. (**a**) Triton Hodge Test of *C. violaceum* M23796 with Meropenem. Black arrow points to outgrowth at the intersection of C. violaceum and the indicator strain indicating the expression of a carbapenemase. White streak to the left is the negative control (C−) and the white streak to the right is the positive control (C+). (**b**) mCIM and eCIM of *C. violaceum* M23796. Disc at the left: Carbapenemase positive with zone diameter of 6mm. The purple border is *C. violaceum* excess growth. Disc at the right: Metallo-β-lactamase positive eCIM with ≥ 5mm increase in diameter zone vs zone diameter for mCIM.
